# Concurrent Diabetes Mellitus may Negatively Influence Clinical Progression and Response to Androgen Deprivation Therapy in Patients with Advanced Prostate Cancer

**DOI:** 10.3389/fonc.2015.00129

**Published:** 2015-06-15

**Authors:** Jeffrey Shevach, Emily Jane Gallagher, Teena Kochukoshy, Victoria Gresia, Manpreet Brar, Matthew D. Galsky, William K. Oh

**Affiliations:** ^1^Division of Hematology and Medical Oncology, Tisch Cancer Institute, Icahn School of Medicine at Mount Sinai, New York, NY, USA; ^2^Division of Endocrinology, Diabetes and Bone Diseases, Department of Medicine, Icahn School of Medicine at Mount Sinai, New York, NY, USA

**Keywords:** androgen deprivation therapy, diabetes mellitus, time to castration-resistant prostate cancer, insulin resistance and cancer, hormone-sensitive prostate cancer

## Abstract

**Objective:**

To determine if a concurrent diagnosis of diabetes mellitus is associated with worse outcomes in advanced prostate cancer (PC). The effect diabetes may have on the progression of advanced PC is poorly understood.

**Methods:**

Data on 148 advanced PC patients (35 with concurrent diabetes) were collected from an institutional database to obtain diabetic status, data on treatment types and durations, and prostate-specific antigen (PSA) values before, during, and after treatment. Time to castration resistance following the onset of androgen deprivation therapy (ADT) and overall survival (OS) in patients with and without diabetes were compared using univariate Cox regression analyses as the primary endpoints. Differences in PSA response to treatments were compared using chi-squared tests as a secondary endpoint.

**Results:**

With a median follow-up of 29 months, time to castration resistance did not differ significantly between patients with and without diabetes who underwent ADT. However, in a subset of patients who received ADT without radiographic evidence of metastases (*N* = 47), those with diabetes progressed to castration-resistant disease more quickly than those without DM (hazard ratio for progression with diabetes = 4.58; 95% CI: 1.92–10.94; *p* = 0.0006). Also, a lower percentage of patients undergoing ADT with diabetes had PSA declines of at least 50% (*p* = 0.17) and reached a nadir PSA <0.2 ng/mL (*p* = 0.06). OS did not differ based on diabetic status. No differences were seen in response to first-line therapy for castration-resistant prostate cancer.

**Conclusion:**

Diabetes mellitus may have a detrimental effect on progression of advanced PC, particularly in those patients without radiographic evidence of metastases. Further study is necessary to fully elucidate the effect of diabetes on PC outcomes.

## Introduction

Previous studies have suggested intriguing associations between prostate cancer (PC) and diabetes mellitus. Examples of such associations include the tendency of PC treatments to induce or exacerbate metabolic syndrome and type 2 diabetes in patients undergoing androgen deprivation therapy (ADT) for advanced PC (1–5). Additionally, some have suggested that diabetes may be a protective factor against developing PC (6–10). Studies have also shown that diabetes and worse glycemic control are associated with higher Gleason scores at initial biopsy and increased mortality (11–14).

The metabolic syndrome and the early stages of type 2 diabetes are frequently associated with insulin resistance in metabolic tissues and a compensatory endogenous hyperinsulinemia in order to maintain normal circulating glucose levels (15). Chronic endogenous hyperinsulinemia has been proposed to be a contributing factor to the progression of PC. Ma et al. found that higher concentrations of C-peptide, a serum marker that reflects endogenous insulin release, were associated with a higher risk of PC mortality (16). Insulin and its binding to the insulin receptor have been shown to activate mitogenic pathways in PC cell lines (17). It has also been demonstrated that insulin increases *de novo* steroidogenesis in PC cells, and that there are increased levels of insulin receptors on human PC cells compared to normal prostatic epithelium, suggesting that insulin is a mediator of PC growth and function (18, 19). But while the current literature analyzes the effects of diabetes on PC, it mostly reflects outcomes in local PC or does not specify disease state at all (20–22).

The effect that diabetes might have on PC progression, especially in advanced disease, is not well studied. In a comprehensive review analyzing the effects of diabetes on PC, Snyder et al. noted that one of their more important findings was the *lack of research on the subject* (23). The gap in the literature is particularly troublesome when considering the epidemiologic overlap between these two common diseases. According to the American Diabetes Association, 27% of the US population over 65 years of age has diabetes, the majority of whom have type 2 diabetes, while data from the SEER PC database indicate that 36.3% of new PC diagnoses are made in men between the ages of 65 and 74. Additionally, a European study found that 32% of their patients with castration-resistant prostate cancer (CRPC) also had a concurrent diagnosis of diabetes (24). This has important clinical implications, considering the findings that insulin can be a growth factor and can increase *de novo* steroidogenesis, and could therefore provide a mechanism for castration resistance. CRPC carries a poor prognosis, with median survivals of patients ranging from 9 to 30 months (25).

In this retrospective study, we examine the correlation between a diagnosis of diabetes mellitus and differences in overall survival (OS), time to castration resistance, and prostate-specific antigen (PSA) response to ADT in men with advanced PC.

## Materials and Methods

### Data collection

Data for this retrospective study were collected from the Icahn School of Medicine at Mount Sinai’s Genitourinary Cancer Biorepository. The study was approved by the Mount Sinai Institutional Review Board (GCO# 10-1180). This research database contains records of date of birth, date of death, race, last known disease status, last known treatment status, start and end dates for treatments, clinical indications for treatments, extent of disease at initial diagnosis, GS at initial biopsy, PSA values and their corresponding dates, and major comorbidities. The database has patient data collected retrospectively to 1991 and is continually updated to ensure quality control.

### Case selection

**Figure 1:**
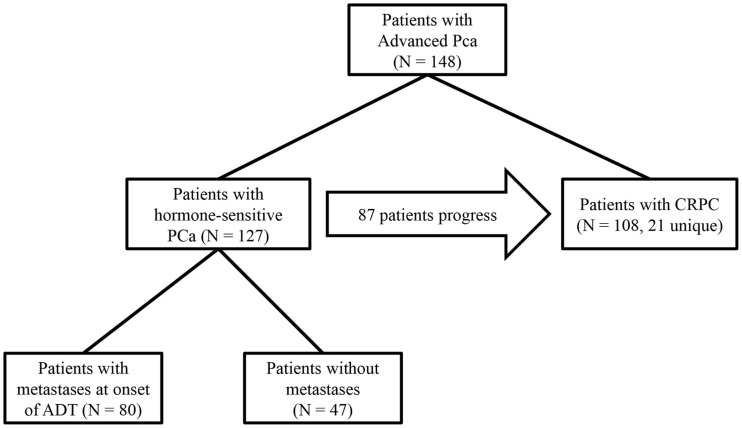
**Study design: 127 hormone-sensitive patients in the database were treated with ADT for advanced PC**. Of those patients, 47 began treatment without radiographic evidence of metastases, 87 hormone-sensitive patients progressed to CRPC, and an additional 21 patients were treated for CRPC whose clinical data from their hormone-sensitive state was not available, totaling 108 patients with CRPC in the study. In all subset of patients, outcomes were compared between patients with diabetes and those without diabetes.

In screening subjects for this study, we only considered patients in the GU Cancer Biorepository who had provided consent to research projects. Preliminary inclusion was based on treatment and disease status; only patients who had begun to receive ADT for non-adjuvant or non-neoadjuvant treatment in the setting of biochemical recurrence or metastatic disease or who received therapy for CRPC were eligible. If a patient progressed from hormone-sensitive PC to CRPC, then the patient was grouped into a study of each respective disease state. Patients with small cell carcinomas of the prostate were not included. A diagnosis of diabetes was determined by the patient history reported either in the database or in their electronic medical record at the Icahn School of Medicine at Mount Sinai. If a patient developed diabetes after the start of their treatment, they were included in the diabetic cohort. A total of 148 patients were enrolled in this study based on the above criteria, many of whom were included in both the studies on hormone-sensitive PC and CRPC (Figure 1). For certain outcome measurements, more data elements were required, and patients were excluded from these measurements if necessary data elements were unavailable. The necessary data elements for each test are included in the results section.

### Outcomes measured and statistical analysis

In the hormone-sensitive cohort, primary outcomes measured were time to CRPC and OS following the onset of ADT. In the cohort of patients with CRPC, the primary outcome was OS following the onset of castration resistance. Secondary outcomes were percentage of patients with a PSA response to ADT or first-line therapy for CRPC as measured by ≥50% reduction, and percentage of patients responding with a nadir PSA <0.2 ng/mL following ADT.

Both time to CRPC and OS were compared between patients with and without diabetes using Kaplan–Meier survival plots and Cox proportional hazard ratios. The difference in the percentage of patients who responded to their treatment based on the percentage of PSA reduction and nadir PSA <0.2 ng/mL between the groups of patients with and without diabetes was compared using the χ^2^ test. All statistics including statistical significance values were computed using Statistical Package for the Social Sciences (SPSS) (26).

### Definition of castration resistance

Time to CRPC was calculated as the time from the onset of non-adjuvant ADT to the date of castration resistance. The following four definitions were used to determine castration resistance, the earliest of which to occur was used as the date of progression: (1) two rises in PSA at least 1 week apart while on castration therapy or after bilateral orchiectomy signified CRPC, and the date of progression used was the date of the first rise. The first rise in PSA must have been at least 0.02 ng/mL greater than the previous PSA value, and the second rise must have been greater than the first rise in PSA, (2) treatment with an androgen receptor analog such as bicalutamide or enzalutamide at least 2 weeks after the start of castration therapy, or switching analogs at least 2 weeks after the start of castration therapy if the initial therapy was combined androgen blockade, (3) treatment with an adrenal steroid enzyme inhibitor (ketoconazole, abiraterone), sipuleucel-T, or non-adjuvant chemotherapy (docetaxel, cabazitaxel), (4) presence of a doctor’s note in the patient’s medical record indicating the patient had CRPC.

### Definition of PSA response

In order to be eligible for analysis of PSA response, patients had to have a baseline PSA measurement within 1 year prior to the onset of treatment type in addition to at least one PSA measurement after the onset of the first treatment but before the onset of additional therapy, the lowest of which was used to calculate the percentage reduction in PSA in response to the given therapy. To be eligible for the analysis of nadir PSA following ADT, patients needed to have PSA values after the onset of ADT and before they progressed to CRPC.

## Results

### Time to CRPC

**Figure 2:**
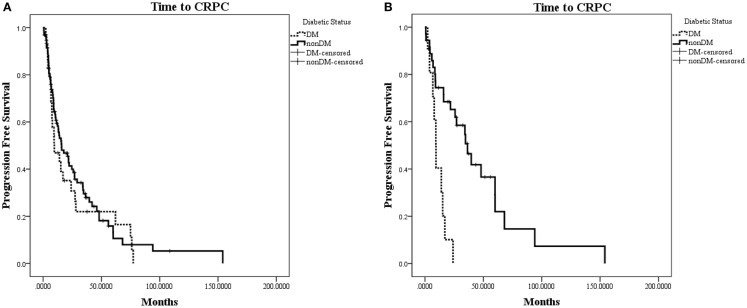
**Kaplan–Meier plot of time to CRPC from onset of ADT**. (**A**) Time to CRPC in all hormone-sensitive patients (*N* = 127). There was no difference between patients with diabetes or without diabetes (*p* = 0.46). (**B**) Time to CRPC in patients without radiographic evidence of metastases (*N* = 47). Patients with diabetes in this subset were observed to progress to CRPC significantly faster than patients without diabetes (HR for progression with diabetes = 4.58; 95% CI: 1.92–10.94; *p* = 0.0006).

The characteristics of eligible patients are detailed in Table 1. Of the 148 patients in the database with advanced PC, 127 underwent ADT for biochemically recurrent or metastatic PC; of these, 30 patients had concurrent diabetes. Median follow-up was 29 months (range 0–154 months). In this cohort, there was no observed difference in time to CRPC between patients with and without diabetes (HR for progression with diabetes = 1.19; 95% CI: 0.77–1.89; *p* = 0.46; Figure 2A).

**Table 1:** **Characteristics of eligible patients**.

Characteristic	Without DM	With DM
	*N* = 113	*N* = 35
Age at start of therapy (years)
Median	68	73
Interquartile range	63-75	66-78
Race (%)
White	70 (61.9)	20 (57.1)
Black	23 (20.4)	7 (20.0)
Hispanic	9 (8.0)	4 (11.5)
Other	8 (7.1)	2 (5.7)
Not reported	3 (2.6)	2 (5.7)
Gleason score (%)
≤7	37 (32.7)	10 (28.6)
8–10	56 (49.6)	19 (54.3)
Missing data	20 (17.7)	6 (17.1)
Metastatic disease at start of ADT (%)
Yes	71 (62.8)	22 (62.9)
No	42 (37.2)	13 (37.1)
PSA level at start of therapy (ng/mL)
Median	18.33	25.39
Interquartile range	5.1–83	3.03–71.14

When patients were stratified based on whether they had radiographic evidence of metastatic disease at the onset of ADT, we observed a greater difference in time to CRPC in the subset of patients without radiographic evidence of metastatic disease (*N* = 47; Figure 2B). In this subset of patients with a median follow-up of 19.7 months, 10 out of 11 (90.9%) patients with diabetes progressed to CRPC, while 24 out of 36 (66.7%) of patients without diabetes progressed to CRPC. The median time to CRPC was 9.4 months for patients with diabetes compared with 36.4 months for those without diabetes (HR for progression with diabetes = 4.58; 95% CI: 1.92–10.94; *p* = 0.0006). There was no difference in time to CRPC between patients with and without diabetes in the subgroup of patients who had metastatic disease at the onset of ADT (HR = 0.65; 95% CI: 0.35–1.19; *p* = 0.16).

### Overall survival

In the same cohort of 127 patients who had undergone ADT for hormone-sensitive PC, 6 out of 30 (20%) patients with concurrent diabetes had died, and 34 out of 97 (35.1%) patients without diabetes died. No statistically significant difference was seen in OS from the time of ADT onset between patients with and without diabetes. Median OS in patients with diabetes was 130.3 months compared with 76.7 months in patients without diabetes (HR for death with diabetes = 0.71; 95% CI: 0.29–1.63; *p* = 0.39).

In 108 patients with CRPC, 25 of whom had diabetes, there was also no significant difference in OS from the date of CRPC diagnosis. Median follow-up after castration resistance was 23 months (range 0–127). Seven out of the 25 patients with diabetes died (28%), and 34 out of the 83 patients without diabetes died (41%). The median survival following the onset of CRPC in patients with diabetes was 84.0 months compared with 35.7 months in patients without diabetes (HR for death with diabetes = 0.57; 95% CI: 0.25–1.30; *p* = 0.184).

### PSA response rate and nadir PSA

A total of 92 patients who underwent ADT were evaluable for PSA response. Patients without diabetes (*N* = 71) trended toward a greater response rate than those without diabetes, though this did not achieve statistical significance (84.5–71.4%; χ^2^ = 1.84; *p* = 0.17). Of 81 patients with CRPC with data available, there was no observed difference in response rate based on a concurrent diagnosis of diabetes (41.3–50%; *p* = 0.51).

A total of 103 patients were eligible for analysis of nadir PSA. About 35.9% of patients without diabetes (*N* = 78) reached a nadir PSA below 0.2 ng/mL compared with 16% of patients with diabetes (*N* = 25), a trend that did not reach statistical significance (χ^2^ = 3.50; *p* = 0.061).

## Discussion

There is a current dearth of literature examining the effects of diabetes on the progression of advanced PC. We tested the hypothesis that a concurrent diagnosis of diabetes would lead to shorter time to CRPC, shorter OS, and worse responses to therapies in patients with PC. Though there was no difference in time to CRPC or OS between patients with and without diabetes in the whole study population, a subset analysis of patients without radiographic evidence of metastases – presumably presenting with biochemical recurrence – revealed a statistically significant difference in time to CRPC between patients with and without diabetes, strongly favoring patients without diabetes. In this study population, all patients with metastases were observed to have a median time to CRPC of 12.4 months, which is consistent with the results from the STAMPEDE trial (27). The effect of diabetes on time to CRPC may have only been observed in patients without metastases at the onset of ADT because these are patients who would otherwise have the potential for a longer progression-free survival, whereas those with metastases have disease that would progress more quickly regardless of diabetic status.

Additionally, PC patients with diabetes were observed to have a trend toward inferior PSA responses following ADT, though these trends did not meet statistical significance. A lower percentage of patients with diabetes experienced a 50% or greater reduction from baseline PSA following ADT, and a lower percentage of patients with diabetes reached a nadir PSA <0.2 ng/mL following ADT, though neither of these observations reached statistical significance. Higher PSA response rates and nadir PSA levels <0.2 ng/mL have each been associated with improved outcomes in patients with advanced PC (28–31). The fact that these indicators trended toward worse outcomes in patients with diabetes was of interest, since it followed the same directional pattern as time to CRPC in non-metastatic PC.

Diabetes is associated with lower levels of testosterone, which is one reason why the adverse effects of ADT include risk of diabetes and hyperglycemia (32–35). With our findings, we speculate that patients with long-standing diabetes might respond more poorly to ADT since their cancers may have developed in the setting of lower testosterone levels, possibly making their disease more castration-resistant. Additionally, new onset diabetes secondary to ADT can provide a mechanism for disease modification and castration resistance, since new onset diabetes is associated with hyperinsulinemia and insulin directly induces *de novo* steroidogenesis in PC cells.

Apart from being a retrospective analysis, the largest limitation of this study was its small sample size, particularly in the diabetic cohort. Additionally, some of the patient data, such as Gleason score at initial biopsy, disease burden at the start of treatment and baseline PSA were unavailable, which lowered our sample and limited our ability to account for potential disease-modifying confounders. For instance, obesity has been shown to influence PC progression in some populations, and is also associated with higher insulin levels and lower testosterone levels (36, 37). Obesity would therefore overlap with our proposed mechanisms for disease progression conferred by diabetes. However, many of our patients did not have data necessary to determine BMI. Additionally, our median follow-up for patients from the onset of ADT was only 29 months. Though this was enough follow-up to observe a trend in time to CRPC, it may have been too short to make definitive conclusions regarding survival.

## Conclusion

These preliminary findings suggest there may be negative effects of concurrent diabetes on progression of advanced PC. Further efforts to fully describe these interactions should include investigation of larger databases for the relationship between diabetes and time to CRPC and OS in patients with advanced PC. Additionally, the observation that the subset of patients without metastases is particularly influenced by diabetes with a shorter time to CRPC needs validation. The effects of glycemic control and reducing endogenous insulin levels on the aforementioned outcomes also require further examination, as targeting these metabolic abnormalities may prove to be an important adjuvant clinical intervention in these patients.

## Author Contributions

JS helped design the study, collected and analyzed the data, and wrote the majority of the manuscript. EG primarily aided in clinical analysis of the data and helped wrote the manuscript. TK, VG, and MB were instrumental in collecting the data and helped make revisions to the manuscript. MG and WO primarily aided in the direction of the study design and helped revise the manuscript. All authors approved of the manuscript submission.

## Conflict of Interest Statement

The authors declare that the research was conducted in the absence of any commercial or financial relationships that could be construed as a potential conflict of interest.
